# Sodium-Glucose Cotransporter-2 Inhibitors in Heart Failure: Role of Regional/Ethnic Differences: Focus on Asia∗

**DOI:** 10.1016/j.jacasi.2023.11.010

**Published:** 2024-02-06

**Authors:** Giulia Crisci, Andrea Salzano

**Affiliations:** a Department of Translational Medical Sciences, Federico II University, Naples, Italy; b Cardiology Unit, AORN A Cardarelli, Naples, Italy

**Keywords:** Asia, dapagliflozin, ethnicity, heart failure, heart failure with mildly reduced ejection fraction, heart failure with preserved ejection fraction, regional differences, sodium-glucose cotransporter-2 inhibitor

**Figure:**
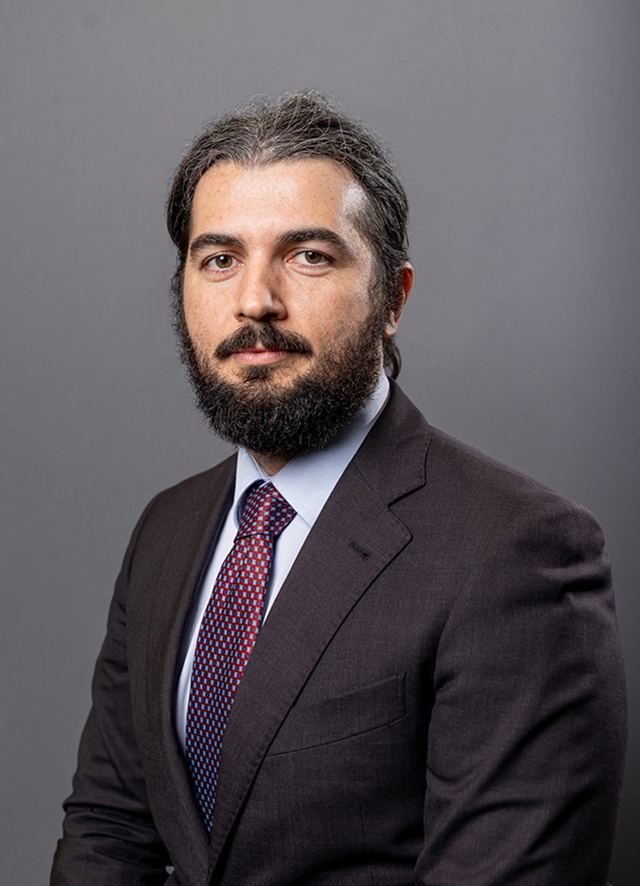

The global burden of heart failure (HF), which has an estimated prevalence of 1% to 3%^1^ and an increasing incidence assessed at 1 to 20 cases per 1,000 person-years,^1^ severely affects health care systems worldwide. Several factors contribute to this ominous trend, such as the progressive aging of population, the improvement of medical therapy, and the increased survival of diseases representing the more frequent etiologic causes of HF (eg, ischemic heart disease).^2^ In this context, the knowledge of specific HF epidemiology across individual global regions still sits within a large evidence gap. Nonetheless, a growing body of evidence shows an increase in the incidence and prevalence of HF in Asia.^3^ Specifically, local registries or trials report very heterogenous data, with HF prevalence ranging from 0.4 to 6.7% (ie, 1.3%-6.7% in South Asia-India, Pakistan, Bangladesh, Sri Lanka, Nepal, Bhutan, and Maldives,^4^ 6% in Taiwan, 5% in Indonesia, 2%-3% in Hong Kong, 1%-2% in Philippines, 1.3% in China,^5^ <1% in Japan, 0.6% in South Korea, and 0.4% in Thailand^6^).

Despite this epidemiologic growth, it has recently been shown that the rate of enrollment of participants from the Asia and Asian-Pacific (APAC) region into randomized clinical trials (RCTs) is low and does not fall in line with the global epidemiology of cardiovascular (CV) diseases.^7^^,^^8^ Specifically, a recent literature review revealed that <15% of participants in HF trials were recruited from APAC.^8^ In addition, a systematic review of all cardiometabolic related RCTs published in the major medical journals in the decade 2011 to 2020 showed that <10% of participants were identified as Asian (a total of 8.3%) and that the total rate of enrollment within the APAC region was only 7.7%.^7^

In the era of big-data trials, this topic is of utmost importance. It has been demonstrated across several fields that huge differences related to regionality and ethnicity are present^9^^,^^10^ and that the under-representation—or, even worse, the noninclusion—of specific research populations in RCTs leads to a lack of generalizability, further spreading the health disparities present in low- and middle-income countries.^11^^,^^12^

In this issue of *JACC: Asia*, Wang et al^13^ performed a post hoc subgroup analysis from the DELIVER (Dapagliflozin Evaluation to Improve the Lives of Patients with Preserved Ejection Fraction Heart Failure) trial that aimed to describe the clinical characteristics, safety, and efficacy of dapagliflozin in patients with heart failure with preserved ejection fraction (HFpEF) and heart failure with mildly reduced ejection fraction (HFmrEF) enrolled within Asia.^14^ As a result, patients enrolled within Asia (19.6% of the total population, all Asians), compared with patients enrolled outside Asia (<1% Asian), demonstrated a lower rate of comorbidities (ie, obesity, dyslipidemia, type 2 diabetes, hypertension, myocardial infarction, stroke, chronic obstructive pulmonary disease, and sleep apnea). Even if a similar incidence of the primary composite endpoint (worsening HF: defined as HF hospitalization or urgent HF visit or CV death) was observed, Asian patients displayed a lower risk of CV death and all-cause death, with some intraregional differences (eg, Japanese patients had a lower risk compared with patients enrolled from China and Taiwan). Overall, the DELIVER study demonstrated that dapagliflozin reduced the combined risk of worsening heart failure or CV death.^13^ However, the present post hoc analysis was not able to show a significant positive effect of dapagliflozin compared with a placebo for patients from Asia; this finding can be explained by the lower number of participants and lower number of events occurring within the Asian subgroup. This limits statistical power to assess the treatment effect of dapagliflozin in this population. Nonetheless, the authors showed that enrollment within Asia did not alter the effect of dapagliflozin on primary outcome (*P* _interaction_ = 0.54), components of the primary outcome, or secondary outcomes (*P*_interaction_ > 0.32 for all outcomes).

To put these findings into context, it is important to highlight that despite the availability of some data in HFrEF from both the EMPEROR-Reduced (Empagliflozin Outcome Trial in Patients with Chronic Heart Failure and a Reduced Ejection Fraction) and the DAPA-HF (Dapagliflozin and Prevention of Adverse Outcomes in Heart Failure) trials,^15^^,^^16^ this investigation is the first complete study focusing on regional differences of patients enrolled from Asia on the effect of sodium-glucose cotransporter-2 (SGLT-2) inhibitors (ie, dapagliflozin) in patients with HFpEF and HFmrEF.

In the EMPEROR-Reduced trial (ie, empagliflozin in HFrEF) the rate of primary events (CV death or HF hospitalization) was the highest in patients enrolled in Asia (13.2% of the total population)^15^; however, these regional differences were mostly driven by differences in the incidence of HF hospitalization, with no differences in CV death. In addition, the magnitude of the effect of empagliflozin on the primary composite outcome and total hospitalizations for HF was most pronounced in patients from Asia and in patients with Asian ethnicity, even when adjusted for confounders. Focusing on the DAPA-HF trial (ie, dapagliflozin in HFrEF), in which 23.1% of the overall population enrolled were from Asia, Asian patients were reported to be younger, more frequently male, and with reduced prevalence of comorbidities (ie, coronary artery diseases, hypertension, obesity, chronic kidney diseases, and atrial fibrillation).^16^ Regarding the primary outcome, patients from Asia had a similar risk for the primary composite outcome of a worsening HF event or cardiovascular death to patients enrolled outside of Asia. In this trial, the authors showed that dapagliflozin reduced the risk of worsening HF or cardiovascular death to the same extent in patients enrolled within Asia as those enrolled outside Asia, with no interaction between geographic region and effect of treatment. However, when specific Asian regions were considered (ie, East Asia vs South Asia vs South East Asia) the effects of dapagliflozin on the primary endpoint was statistically significant only for the East Asian population.^16^

Beside HFrEF trials, preliminary data from patients with HFpEF are now available; specifically, Chopra^17^ (on behalf of the EMPEROR-Preserved investigators) presented data at the American College of Cardiology 2022 conference (Washington, DC, USA), demonstrating that no interaction for treatment effect of empagliflozin by region or race was observed for the primary endpoint (ie, cardiovascular death or HF hospitalization), time to first HF hospitalization, or time to CV death. In addition, even though the exploratory analysis by region indicated heterogeneity for HF hospitalization, the analysis by race did not.

In conclusion, the present investigation^14^ adds to the knowledge of the effects of SGLT-2 inhibitors (ie, empagliflozin and dapagliflozin) in patients with HF and, specifically, on patients enrolled in Asian countries (Figure 1). Indeed, overall data from the present and other analyses of the main RCTs (Table 1) suggest that enrollment from Asia does not modify the efficacy of SGLT-2 inhibitors in patients with HF and that they are safe and effective in Asian patients as well as in non-Asian patients. With regard to the effects, all the available trials align in showing an association of the use of SGLT-2 inhibitors with reduction of primary outcomes, even in patients enrolled in Asia, with the only exception being the present post hoc analysis, which was limited by statistical power. However, if the present post hoc analysis does not reduce the importance and the impact of the DELIVER trial, it does strongly focus attention on the imperative need to increase the representation of patients from Asia (and from other countries worldwide) in the enrollment of clinical trials, pointing out the need of ethnic and regional equality in the design of large trials, and that international scientific societies and drug regulatory authorities should be involved in this process to reduce ethnic and regional inequality.

**Figure 1:**
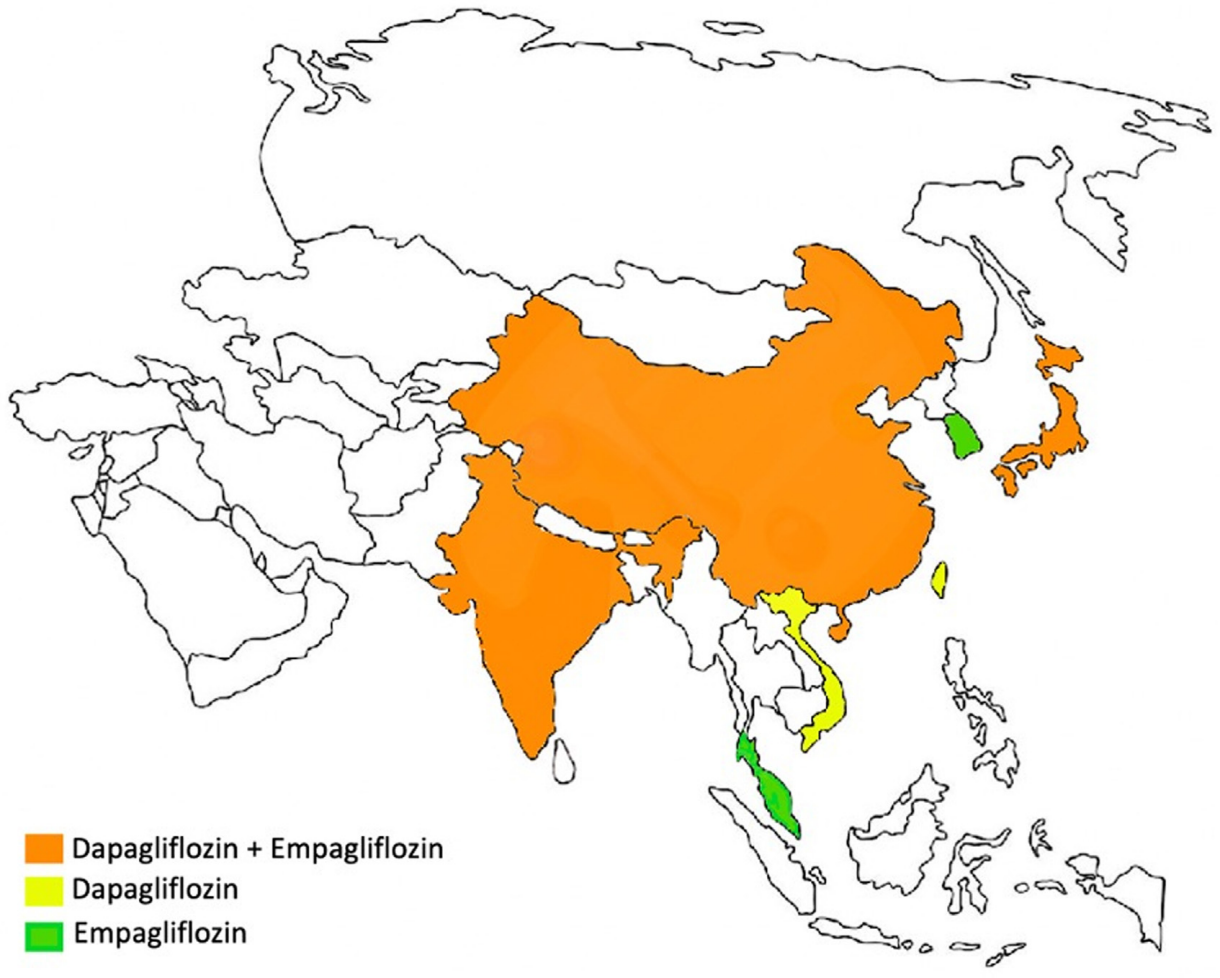
SGLT-2 Inhibitors in Heart Failure in Asia The availability of data regarding the use of sodium-glucose cotransporter-2 (SGLT-2) inhibitors in heart failure across Asian countries.

**Table 1:** Summary of Evidence From the Registrative Randomized Control Trials of SGLT-2 Inhibitors and Heart Failure

HF Phenotype	Trials	Patients Enrolled From Asia (x di Y; Z%)	Main Findings
HFrEF	DAPA-HF^16^	1,096 of 4,744; 23.1%	Primary composite endpoint: Dapagliflozin reduced the risk of worsening HF (HHF or urgent HF visit) or CV death in Asia and outside Asia. The risk of a worsening HF, CV death and all-cause death are similar in Asian and outside Asian patients. Additional comment: Several secondary endpoints were consistent with results in patients enrolled outside Asia.
	EMPEROR-Reduced^15^	672 of 3,730; 18%	Primary composite endpoint: • Reduction in CV death or HHF in Asia were more pronounced than in North/Latin America and Europe. • Total hospitalizations for HF was most pronounced in Asia, followed by North/Latin America or Europe.
HFpEF/HFmrEF	Deliver-HF^14^	1,226 of 6,263; 19.6%	Primary composite endpoint: • Dapagliflozin reduced the risk of worsening HF (HHF or urgent HF visit) or CV death in participants from both Asia and outside Asia Enrollment within Asia had an almost 50% lower risk of CV death, non-CV death, and all-cause death
	EMPEROR-Preserved^17^^a^	686 of 5,988; 11,5%	Primary composite endpoint: • Dapagliflozin reduced the risk of worsening HF (HHF or urgent HF visit) or CV death in participants from both Asia and outside Asia No difference of HHF between patients from Asia and outside Asia

CV = cardiovascular; DAPA-HF = Dapagliflozin and Prevention of Adverse Outcomes in Heart Failure trial; DELIVER-HF = Dapagliflozin Evaluation to Improve the Lives of Patients with Preserved Ejection Fraction Heart Failure; EMPEROR-preserved = Empagliflozin Outcome Trial in Patients with Chronic Heart Failure and a Preserved Ejection Fraction; EMPEROR-Reduced = Empagliflozin Outcome Trial in Patients with Chronic Heart Failure and a Reduced Ejection Fraction; HF = heart failure; HFmrEF = heart failure with mildly reduced ejection fraction; HFpEF = heart failure with preserved ejection fraction; HFrEF = heart failure with reduced ejection fraction; HHF = hospitalization for heart failure; SGLT-2 = sodium-glucose cotransporter-2.

a Only preliminary data are available.

## Funding Sources and Author Disclosures

Dr Crisci has received a research grant from the CardioPath program, Federico II University, Naples, Italy. Dr Salzano has reported that he has no relationships relevant to the contents of this paper to disclose.
